# Single, Dual, and Triple Use of Cigarettes, e-Cigarettes, and Snus among Adolescents in the Nordic Countries

**DOI:** 10.3390/ijerph19020683

**Published:** 2022-01-07

**Authors:** Kirsimarja Raitasalo, Elin K. Bye, Charlotta Pisinger, Janne Scheffels, Rikke Tokle, Jaana M. Kinnunen, Hanna Ollila, Arja Rimpelä

**Affiliations:** 1 Department of Public Health and Welfare, Finnish Institute for Health and Welfare, 00271 Helsinki, Finland; hanna.ollila@thl.fi; 2 Department of Alcohol, Tobacco and Drugs, Norwegian Institute of Public Health, 0213 Oslo, Norway; elinkristin.bye@fhi.no (E.K.B.); janne.scheffels@fhi.no (J.S.); rikketok@oslomet.no (R.T.); 3 Center for Clinical Research and Prevention, Bispebjerg and Frederiksberg Hospital, 2000 Frederiksberg, Denmark; charlotta.pisinger@regionh.dk; 4 Department of Public Health, University of Copenhagen, 1353 Copenhagen, Denmark; 5 Danish Heart Foundation, 1120 Copenhagen, Denmark; 6 Norwegian Social Research, Oslo Metropolitan University, 0170 Oslo, Norway; 7 Unit of Health Sciences, Faculty of Social Sciences, Tampere University, 33014 Tampere, Finland; jaana.kinnunen@tuni.fi (J.M.K.); arja.rimpela@tuni.fi (A.R.); 8 Department of Adolescent Psychiatry, Tampere University Hospital, 33521 Tampere, Finland

**Keywords:** multiple use, cigarettes, electronic cigarettes, snus, Nordic countries

## Abstract

New tobacco and nicotine products have emerged on the market in recent years. Most research has concerned only one product at a time, usually e-cigarettes, while little is known about the multiple use of tobacco and nicotine products among adolescents. We examined single, dual, and triple use of cigarettes, e-cigarettes, and snus among Nordic adolescents, using data of 15–16-year-olds (n = 16,125) from the European School Survey Project on Alcohol and other Drugs (ESPAD) collected in 2015 and 2019 from Denmark, Finland, Iceland, Norway, Sweden, and the Faroe Islands. Country-specific lifetime use of any of these products ranged between 40% and 50%, and current use between 17% and 31%. Cigarettes were the most common product in all countries except for Iceland, where e-cigarettes were remarkably more common. The proportion of dual and triple users was unexpectedly high among both experimental (24%–49%) and current users (31–42%). Triple use was less common than dual use. The users’ patterns varied somewhat between the countries, and Iceland differed substantially from the other countries, with a high proportion of single e-cigarette users. More knowledge on the patterns of multiple use of tobacco and nicotine products and on the potential risk and protective factors is needed for targeted intervention and prevention efforts.

## 1. Introduction

Adolescent cigarette smoking has declined in the Nordic countries as well as in many other European countries since the turn of the millennium [1,2], while e-cigarette use has emerged during the last decade [3,4,5]. The emergence of e-cigarettes on the market has subsequently contributed to changing patterns in the use of tobacco and nicotine products, particularly, to the multiple use of these products [6,7]. Even though the health impact on the general population of the multiple use of these products is still unknown [8], the growing use of multiple products among adolescents, shown particularly in the U.S.A., has become a subject of public health concern [9,10]. 

E-cigarettes have been marketed for smoking cessation for adults, but they have also raised interest among adolescents. In the U.S.A., a recent study showed that almost every fifth high school student and 5% of middle school students reported current e-cigarette use [11]. In Europe, a seven-country study showed a high variation in monthly e-cigarette use among 14- to 17-year-olds in 2016–2017, with an average of 6.6% [12], while the ESPAD survey (the European School Survey Project on Alcohol and other Drugs) a few years later showed that the European average of e-cigarette use in the last 30 days was 14% among 15- to 16-year-olds [1]. An Icelandic trend study also showed an exponential increase in ever, current, and daily e-cigarette use among 13–16-year-olds from 2015 to 2018, while cigarette smoking remained mostly unchanged [13]. 

The age limit for selling and buying e-cigarettes is 18 years in all Nordic countries. Otherwise, the strictest regulations have been imposed in Norway, which is only now processing regulations that allow nicotine-containing e-liquids in domestic shops. Of the countries with these products on sale, Finland has imposed the strictest regulations, as e-cigarettes have been subject to retail licensing, a full advertising ban, a point-of-sale display ban, and a ban on non-tobacco flavors in the e-liquids since 2016. Additionally, both nicotine-containing and nicotine-free e-liquids are subject to taxation in Finland and Sweden, and Denmark will introduce a tax in 2022. Denmark has adopted plain packaging for e-cigarettes, which will enter into force in 2022. In Finland, if the current legislative proposal will be adopted, plain packaging for e-cigarettes and e-liquids will enter into force in 2023. The e-cigarette regulations have for long been the weakest in Iceland—this country adopted the first national legislation for e-cigarettes in 2018, which entered into force in 2019. Since then, a new governmental bill has been also under consideration proposing stricter regulations of e-liquid flavors [14].

From a public health point of view, it is of concern that adolescents who have never tried conventional cigarettes have reported experimentation with nicotine-containing e-cigarettes [15], meaning a risk for nicotine addiction in addition to other toxic effects of e-cigarettes [16]. Results from several prospective studies, mainly from the U.S.A. but also from Finland, suggest an association between e-cigarette use and subsequent tobacco use [17,18,19,20], and the risk of smoking uptake has been found to be the highest among adolescent e-cigarette users with no intention to smoke at baseline [21,22].

The majority of adolescents who report the use of e-cigarettes are also found to have experience with other tobacco products [1,7,12,15,23]. However, smoking cessation is only rarely mentioned among youth as a reason for using e-cigarettes, and flavors, curiosity, peer influence, and perceptions of e-cigarettes as “cool” and harmless have been reported as the top reasons for experimentation with them [15,24,25,26]. In addition, studies differentiating between using e-cigarettes with and without nicotine have shown that many adolescents report the use of nicotine-free e-cigarettes [12,23].

In addition to conventional cigarettes and e-cigarettes, snus is a commonly used tobacco product in the Nordic countries. In these countries, snus is sold legally only in Sweden and Norway (to customers 18 years of age and over), but it is available for youth in Iceland, Denmark, and Finland through travel imports or illegal channels. In addition, the circumvention of legislation through the sale of nicotine pouches or chewing tobacco has brought new snus-like products to the markets or to the imports [27]. The prevalence of adolescents’ snus use varies between the Nordic countries and has increased during the last decade in Finland, Sweden, and Norway [20,28,29]. While snus use is more prevalent among males than females, it has increased substantially among girls and women during the last two decades in Norway [29], Sweden [28,30] and to some extent also in Finland [20]. Like non-nicotine e-liquids, non-nicotine snus is also on the market, but there are no studies on adolescents distinguishing the prevalence of the use of nicotine-infused snus from that of non-nicotine-infused snus.

Multiple use, defined as use of two or more tobacco or nicotine products, can emerge when several nicotine-containing and tobacco-like products are on the market. Traditionally, research has concentrated on one tobacco or nicotine product at a time, while less is known about the multiple use of nicotine-containing and tobacco-like product in the general population and among adolescents. Some studies investigating patterns of multiple use have found an association between e-cigarette use and use of other tobacco products [7,31,32]. A Norwegian study among adolescent aged 13–17 years showed that 1 in 3 of those who had tried or used tobacco products or e-cigarettes had tried or used two products, and one in ten had tried or used three products [5]. There are recent studies showing that at least half of the adolescents who use tobacco- and tobacco-like product actually use multiple products [12,32,33]. A Finnish study has shown an increasing number of dual users of snus and cigarettes [20]. A recent study from the U.S.A. also found that most adolescents who initiated any nicotine product use had an elevated risk of transitioning to multiple use [34]. Further, older, in contrast to younger, adolescents and boys in contrast to girls more frequently use multiple nicotine products [35,36].

The knowledge of multiple tobacco and nicotine product use among adolescents in the Nordic countries is still scarce. The availability of data from the European School Survey Project on Alcohol and Drugs (ESPAD) provides a unique opportunity to compare the patterns of such use between countries. The present study aims to explore and compare 1) the prevalence of single, dual, and triple use of cigarettes, e-cigarettes, and snus among adolescents in the Nordic countries and 2) to study differences in multiple use between experimental and current users.

## 2. Materials and Methods

### 2.1. Data

Data were obtained from the ESPAD study, where cross-sectional school surveys were conducted every fourth year between 1995 and 2019 to investigate substance use among 15- to 16-year-old students [1]. The present analyses were based on data on the use of e-cigarettes, cigarettes, and snus in six Nordic countries: Denmark (DE), Finland (FI), Iceland (IS), Norway (NO), Sweden (SW), and the Faroe Islands (FO). Denmark, the Faroe Islands, and Sweden did not include questions for all three products in 2019 (snus was missing for all); thus, data from 2015 were used for these countries (n = 4 735), while data from 2019 (n = 11 388) were used for Finland (FI), Iceland (IS), and Norway (NO). An overview of the samples, student participation rates, and prevalence of the different substances is displayed in Supplementary Table S1. 

### 2.2. Measures

Lifetime cigarette use was assessed by asking “On how many occasions (if any) during your lifetime have you smoked cigarettes (excluding e-cigarettes)?”, with seven response categories ranging from 0 to 40+ times. This was dichotomized into 0 = No use and 1 = Lifetime use.

Current cigarette use was assessed by asking “How often have you smoked cigarettes (excluding e-cigarettes) during the last 30 days?”, with seven response categories ranging from “Not at all” to “More than 20 cigarettes per day”. This was dichotomized into 0 = Not at all/no use and 1 = Current use. 

Lifetime and current use of e-cigarettes were assessed by asking “Have you ever used e-cigarettes? (Mark all that apply)”, with four response options: “Yes, in the last 30 days”, “Yes, in the last 12 months”, “Yes, more than 12 months ago”, and “Never”. Those answering “yes” to any of these options were classified as lifetime e-cigarette users, and the others as non-users. Correspondingly, those answering “yes” to use in the last 30 days were defined as current users. 

Lifetime- and current use of snus was assessed by asking “Have you ever used snus? (Mark all that apply)”, with the same four response options as for e-cigarettes and thus dichotomized in the same way into lifetime snus use (yes/no) and current use (yes/no). In Norway, the question was asked differently: “Do you use, or have you ever used snus?”, with four response categories: “No, never”, “Yes, but have stopped now”, “Yes, sometimes”, and “Yes, daily”. Those answering “yes” to any of these options were classified as lifetime snus users, and the others as non-users. Daily use was considered as a proxy for current use (yes/no).

To separate between single, dual, and triple use of the different tobacco and tobacco-like products, we first calculated a variable for any lifetime use, based on the three lifetime use variables. This was further divided into (1) single use of any of the substances, (2) dual use of any of the substances (combination of two of the substances), or (3) triple use (use of all three substances). Those who only reported lifetime use of any of the substances (and no use in the past 30 days), were classified as experimental users. Current users were defined as users of any of the substances in the past 30 days. 

### 2.3. Statistical Analyses 

Descriptive statistics were computed using SAS Enterprise Guide 7.1. For countries where non-proportionate stratification for sampling was used (Denmark, Finland, and Norway), data were weighted to account for sample-specific characteristics [1,37]. Iceland and the Faroe Islands surveyed the whole target group; thus, no weights were needed. 

## 3. Results

**Figure 1:**
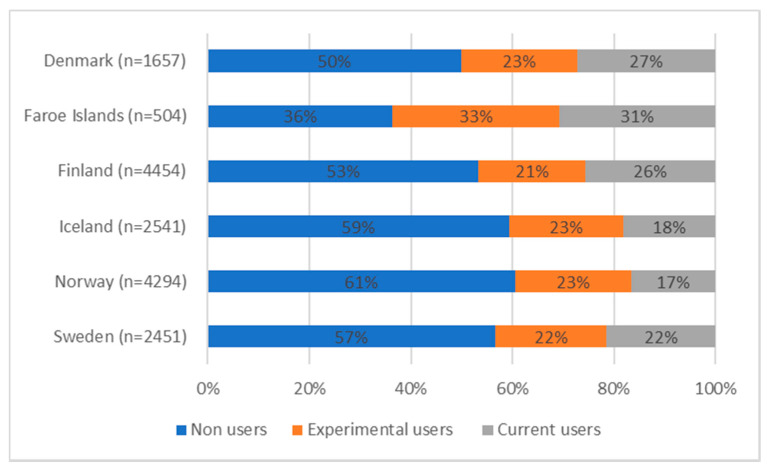
Proportions of students with experimental or current use of at least one tobacco or nicotine product, by country.

Figure 1 shows the proportions of adolescents in each Nordic country with experimental and current use of at least one tobacco or nicotine product. The proportion of those who had never used either cigarettes, e-cigarettes, or snus was the highest in Norway (61%), Iceland (59%), and Sweden (57%), followed by Finland (53%) and Denmark (50%). The Faroe Islands stand out with the lowest number of non-users; only 36% of the participants reported no lifetime use of any of the three substances. Small differences between countries were observed for experimental use (lifetime use but no use during the last 30 days); the prevalence varied between 21% and 23%, except for the Faroe Islands, where the prevalence was 33%. To continue, more variation between countries was observed in current use, which was almost twice as common in the Faroe Islands (31%) compared to Norway (17%) and Iceland (18%).

Table 1 presents the proportions of users of each tobacco or nicotine product by country separately for experimental and current users. The figures include all patterns of use (single, dual, and triple use), and we see that a substantial proportion, ranging from 17 to 31%, of 15–16-year-olds reported current use of at least one tobacco or nicotine product. Among current users, cigarettes were the most commonly used product in all countries except for Iceland, where e-cigarettes were remarkably more common. In Norway, current use of cigarettes and e-cigarettes were equally common. Current use of snus was more common than current use of e-cigarettes in the Faroe Islands, Finland, and Sweden. Among experimental users, the pattern was quite similar with regard to cigarette and e-cigarette use. Experimental use of snus was less common than the use of cigarettes or e-cigarettes among adolescents in all Nordic countries.

**Figure 2:**
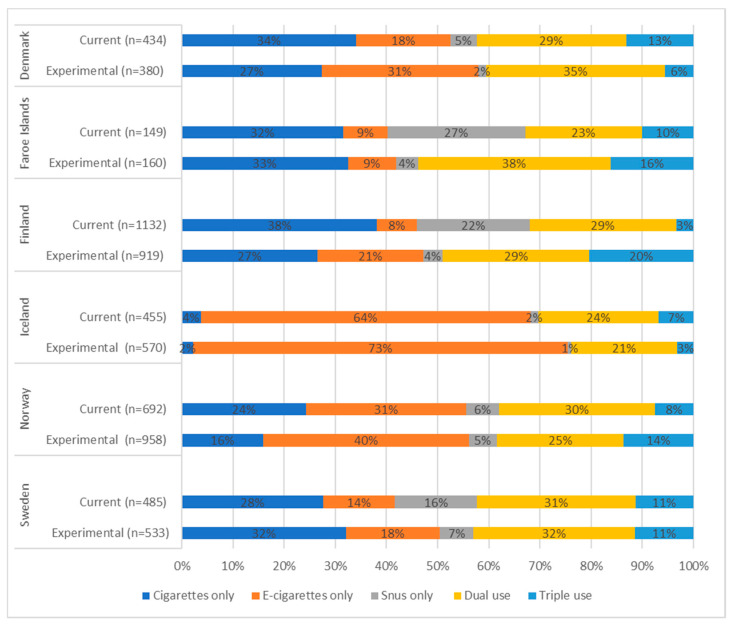
Percentages of lifetime single, dual, and triple use of cigarette, e-cigarette, and snus among current and experimental users, by country.

Figure 2 shows the proportions of single, dual, and triple users among current and experimental users of any tobacco or nicotine products and how these products were used in these user groups by country. Overall, the proportions of single users were higher than the proportions of multiple users (dual and triple users), with the exception of experimental users in the Faroe Islands. However, the proportion of current multiple users was quite substantial, with the highest proportions in Norway (38%) and Sweden (42%) and 31–33% of current multiple users in the other countries. In all countries and in both groups, triple use was less common than single or dual use, varying from 3% in Iceland to 20% in Finland among experimental users and from 3% in Finland to 13% in Denmark among current users.

Moreover, the users’ patterns varied between countries but less so within a country. Iceland differed substantially from the other countries; in both groups, most adolescents used only e-cigarettes (experimental 73%, current 64%), and the use of conventional cigarettes was very low (4% and 2%, respectively). Among experimental users, the proportion of single snus users was low (1%–7%), but among current users, the proportions were higher and particularly so in the Faroe Islands (27%), Finland (22%), and Sweden (16%). The use of conventional cigarettes was the most common pattern of current single use (28%–38%) except in Iceland (4%) and Norway (24%), where the use of e-cigarettes was the most common (64% and 31%, respectively). 

The co-use of cigarettes and e-cigarettes (not shown in Figure 2) was the most common pattern of current dual use in all countries (15%–21%) except, again, in the Faroe Islands, where the co-use of cigarettes and snus was the most common (12%), and in Sweden, where current dual use of cigarettes and snus was as common as dual use of cigarettes and e-cigarettes (both 11%). For experimental users, the results were similar: experimental co-use of cigarettes was the most common pattern of dual use in all countries (11%–32%) except in the Faroe Islands, where experimental co-use of e-cigarettes and snus was the most popular (19%).

## 4. Discussion

The present study is the first to compare patterns of use of cigarettes, e-cigarettes, and snus among adolescents in the Nordic countries. Our findings revealed a substantial proportion of current users of any tobacco or nicotine product as well as high percentages of dual and triple users among 15–16-year-olds. The co-use of cigarettes and e-cigarettes was the most common pattern of current dual use in most countries. The users’ patterns varied somewhat between the countries, and especially, Iceland differed considerably from the other countries, with a very high proportion of single e-cigarette users. Earlier studies have shown connections between use of different tobacco, tobacco-like, and nicotine products among adolescents [12,23,32,33]. Even though the proportions of dual and triple users varied by country in our study, the results show that multiple substance use is common in all Nordic countries. It is difficult to compare our results internationally due to different study populations, different definitions of use, and differences in the included products. However, our result showing that dual and poly-use among adolescents is quite substantial among those who use the products is in line with recent studies from elsewhere in Europe [12,38], Asia [39], and the U.S.A. [40]. It seems that nearly half or even more of the adolescents who use tobacco- or tobacco-like products actually use more than one product.

Adolescents are often open to all new experiences, and curiosity and sensation seeking belong to the developmental phase of that age. When more products on the market are available to adolescents, through either legal or social sources, adolescents are interested in experimenting with them. A recent study also showed that a majority of adolescents who had initiated the use of any nicotine product had an elevated risk of transitioning to poly-use [37]. 

It has been argued that it would be positive that adolescents use next-generation nicotine products instead of conventional cigarettes [35,41,42]. A European study of adolescents [12] suggests, however, that e-cigarettes and snus are complementary to conventional cigarettes and not substitutes, as was suggested earlier. The multiple use of nicotine-containing products increases nicotine exposure and, as a consequence, the risk of dependence in adolescents [43]. A study of Korean adolescents showed that current dual users even smoked more conventional cigarettes per day than smokers only [44]. Users of cigarettes and smokeless tobacco also report more dependence symptoms than those who smoke cigarettes or use smokeless tobacco alone [45]. 

Product availability for minors along with other control measures affect the initiation and regular use of these products. When considering the implementation of the core tobacco control measures of the WHO Framework Convention on Tobacco Control (WHO FCTC), no major differences exist in the regulations of conventional cigarettes [14]. More variation is seen in the regulation of e-cigarettes and snus. For instance, in Sweden and Norway, snus is legally sold, but youth in the other Nordic countries have access to these products through different channels or through the introduction of snus-like products that can circumvent the current legislation. Between Norway and Sweden, there are also differences in the regulation of snus: Norway has imposed plain packaging for snus, while Sweden has not. For e-cigarettes, there is also large variation, from nicotine-containing e-liquids only now being allowed to be sold in domestic shops—which is the case in Norway—to non-existent regulations until recent years, which is the case in Iceland. Additionally, some countries such as Finland and Denmark have adopted regulations to prevent products with specific appeal to youth, such as flavored e-liquids. 

It is difficult to compare the patterns of different product use between countries, as the patterns reflect not only the differences in regulations, but also other factors such as social norms. Snus use among 15–16-year-old adolescents is not particularly high in Norway and in Sweden compared to the other Nordic countries. The regulations imposed on snus and the snus industry in Sweden, which has an exemption in the EU to allow snus sales in the country, seem insufficient to prevent illegal exports to other Nordic countries. E-cigarette use is far above that in the other Nordic countries in Iceland, where the first national legislation on e-cigarettes was adopted only in 2018, entering into force in 2019, which means that minimum age limits, advertising bans, and other protective measures were lacking before that date. In Denmark, where snus is forbidden, chewing tobacco in pouches (very difficult to see a difference from snus) has recently become popular, as the snus ban does not cover this product. A Danish study among 15–29-year-olds found that eight out of ten adolescents/young adults are exposed to snus or chewing tobacco in their daily lives, either by way of friends’ use or by social media, and every third respondent reported to be exposed to tobacco/nicotine product marketing [46]. The novel products that can circumvent the existing regulations, including snus-like products but also heated tobacco products, complicate the preventive efforts further and call for a more unified approach to implement the WHO FCTC measures in the Nordic region.

Control measures are not the only factor in adolescents’ tobacco and nicotine product use. As adults are role models for adolescents, the general pattern in the adult population is important. The youth culture has its own young trendsetters and bloggers who may also influence adolescents’ preferences, e.g., the “straight edge” subculture which—among other things—refers to a lifetime commitment to abstaining from drugs, tobacco, and alcohol. Additionally, the social norms against or pro tobacco or nicotine products vary in time and place. An Icelandic study described a steep increase in the use of e-cigarettes by youth and a continued very low use of conventional cigarettes [13], which we also observed to be the exceptional pattern of Iceland in our study. The change was explained by the strong primary prevention efforts that have been implemented both nationally, by municipalities, and in school communities of Iceland (the latter commonly labelled The Icelandic Model of Primary Substance Use Prevention [47]) over the last 20 years. This has led to a consistent social norm among Icelandic youth that cigarette smoking and other tobacco use are harmful and should be avoided at all costs, whereas norms surrounding the potential harm of e-cigarettes have been less developed in the Icelandic society [13]. Third, tobacco control policies (price and non-price) have been shown to influence adults’ smoking [48], and it is reasonable to assume that they also have an impact not only on adolescents’ smoking habits but also on their choice of nicotine-containing products. However, large differences in adolescent e-cigarette use have also been found in similar regulatory environments [12]. 

Furthermore, e-cigarettes have also contributed to additional changes in the nicotine and tobacco landscape, with the emergence of alternative e-cigarette user practices, whereby the intake of nicotine is not necessarily the main motivation [25,49]. Examples include the use of e-cigarettes to satisfy curiosity, to experience flavors, to conform to peer influences, to perform vape-tricks [50], or to administer cannabis [51].

### Strengths and Limitations 

This study has been conducted on large samples using a common instrument across countries with high response rates, making the data representative of Nordic 15- to 16-year-olds. Furthermore, the data collection was completely anonymous, and all questionnaires were put in individual and unmarked envelopes, which increased the probability of giving truthful responses to sensitive questions.

Still, there are limitations which need to be considered when discussing the results of this study. First, self-reporting data have the risk that students consciously or unconsciously do not give accurate or honest answers about all substances. These incorrect answers may be in both directions, i.e., there may be over-reporting as well as under-reporting depending on what is socially desired/accepted in different contexts. However, a validity report on ESPAD [52] shows that only a very small minority (1–2%) does not answer to questions on substance use honestly. Adolescents’ self-reporting on smoking has also been shown to be accurate and to correspond to biochemical measures [53,54,55]. Secondly, we do not have data on whether e-cigarettes contained nicotine or not. There could be differences in the use of nicotine and non-nicotine e-liquids between the Nordic countries, as differences have been found between European countries [12,23]. Third, we had to use data for different years (2015 and 2019) to be able to compare all Nordic countries. However, as shown in Table S1, the prevalence rates between 2015 in 2019 are very similar for the countries in question, and there have been only small changes in the use of tobacco and e-cigarettes in most Nordic countries in that period [26]. 

## 5. Conclusions

To conclude, we found both similarities and differences in the prevalence of different patterns of use of cigarettes, e-cigarettes, and snus among adolescents in the Nordic countries. Overall, conventional cigarettes were the most popular tobacco or nicotine products among both experimental and current users in all Nordic countries except for Iceland, which stood out with a low smoking prevalence and a high prevalence of e-cigarette use. Although single use was more prevalent than multiple use among both current and experimental users, the proportions of multiple users were remarkably high. This finding calls for attention when planning future prevention efforts. 

To better understand the multiple use of tobacco and nicotine products among adolescents, more knowledge is needed about users’ patterns among both genders and adolescents’ transition between different types of tobacco and nicotine products, such as cigarette smoking, e-cigarette use, and snus use. The nicotine content of e-cigarettes as well as of other novel products should also be included in youth studies, given that the addiction-producing nature of nicotine is one of the most important facts contributing to the popularity of tobacco and nicotine products. Furthermore, it is important that future studies investigate user characteristics, including known predictors of smoking such as sensation seeking, other deviant behaviors, and mental health problems. Knowledge on potential risk factors and common liabilities is important for targeted intervention or prevention efforts.

## Figures and Tables

**Table 1:** Proportions of cigarette, e-cigarette, and snus users among experimental and current users, by country.

	Denmark	Faroe Islands	Finland	Iceland	Norway	Sweden
Current use (last 30 days use)						
	N = 450	N = 155	N = 1144	N = 462	N = 711	N = 528
Cigarettes	71%	62%	68%	28%	60%	61%
E-cigarettes	58%	30%	29%	94%	61%	45%
Snus	27%	52%	39%	15%	25%	47%
Experimental users (lifetime use)						
	N = 380	N = 161	N = 925	N = 570	N = 958	N = 534
Cigarettes	67%	67%	73%	24%	48%	71%
E-cigarettes	69%	59%	64%	97%	72%	55%
Snus	10%	44%	32%	24%	31%	28%

## Data Availability

The ESPAD trend data are archived at the Italian National Research Council (CNR), and data can be used for research purposes.

## Supplementary Materials

The following are available online at https://www.mdpi.com/article/10.3390/ijerph19020683/s1: Table S1: Descriptive statistics of the study population by country and study year.
