# Supplementary material for: Single, Dual, and Triple Use of Cigarettes, e-Cigarettes, and Snus among Adolescents in the Nordic Countries
Source: Int J Environ Res Public Health. 2022 Jan 7;19(2):683. doi: 10.3390/ijerph19020683 (PMC8775390; doi:10.3390/ijerph19020683)
Supplement: Supplementary file 1 [file ijerph-19-00683-s001.zip › ijerph-1515238-supplementary.pdf]

Table S1. Descriptive statistics of the study population, by country, gender and study year.

		***Denmark*[a]**			***Finland***	***Faroe Islands*[a]**			***Iceland***	***Norway***	***Sweden*[a]**		
Study year		2015	2019	$p(\chi^2)$[b]	2019	2015	2019	$p(\chi^2)$[b]	2019	2019	2015	2019	$p(\chi^2)$[b]
Student participation rate, %		88 %	88 %		88 %	92 %	82 %		No info	89 %	86 %	85 %	
Participants, N		1670	2487		4476	511	511		2559	4325	2584	2546	
Gender	Boys	48 %	48 %		50 %	50 %	49%		49 %	50 %	50 %	49%	
	Girls	52 %	52 %		50 %	50 %	51%		51 %	50 %	50 %	51%	
Cigarette use													
Lifetime, %	Boys	37 %	43 %	.004	43%	50 %	57%	ns	16%	29%	33 %	26 %	.000
	Girls	41%	41 %	ns	35%	49 %	34%	.001	14%	21%	34 %	27 %	.000
30 days, %	Boys	17 %	22%	.006	18%	17 %	22%	ns	4%	13%	11 %	10 %	ns
	Girls	21 %	23%	ns	17%	20 %	13%	.044	6%	7 %	14 %	12 %	ns
E-cigarette use													
Lifetime, %	Boys	42 %	44 %	ns	44 %	45 %	53 %	ns	40 %	39 %	33 %	36 %	ns
	Girls	38 %	27 %	.000	24%	43 %	27 %	.000	40 %	22 %	25 %	23 %	ns
30 days, %	Boys	18 %	19 %	ns	9 %	9 %	12 %	ns	15 %	13 %	11 %	9 %	.026
	Girls	14 %	10 %	.003	5 %	9 %	5 %	.044	19 %	7 %	7 %	4 %	.001
Snus use													
Lifetime, %	Boys	19 %	-		35 %	45 %	-		9 %	22 %	29 %	-	
	Girls	8 %	-		19 %	31 %	-		9 %	17 %	13 %	-	
30 days, %	Boys	11 %	-		11 %	21 %	-		3 %	5 %	15 %	-	
	Girls	4 %	-		9 %	10 %	-		2 %	3 %	5 %	-	

[a] For Denmark, Faroe Islands and Sweden, data from 2015 was used.

[b] Chi-square tests were used to assess the statistical significance in the prevalence of different tobacco and nicotine product use between years 2015 and 2019 in countries where data from 2015 was used.

[c] For comparison with other ESPAD countries, see http://espad.org/sites/espad.org/files/20203880_TD0320532ENN_PDF.pdf.
